# Considerations for bioanalytical characterization and batch release of COVID-19 vaccines

**DOI:** 10.1038/s41541-021-00317-4

**Published:** 2021-04-13

**Authors:** Gautam Sanyal, Anna Särnefält, Arun Kumar

**Affiliations:** Vaccine Research and Development, Coalition for Epidemics Preparedness Innovations, CEPI, Torshov, Oslo Norway

**Keywords:** Biotechnology, Immunology, Vaccines

## Abstract

The COVID-19 pandemic has prompted hundreds of laboratories around the world to employ traditional as well as novel technologies to develop vaccines against SARS-CoV-2. The hallmarks of a successful vaccine are safety and efficacy. Analytical evaluation methods, that can ensure the high quality of the products and that can be executed speedily, must be in place as an integral component of Chemistry, Manufacturing, and Control (CMC). These methods or assays are developed to quantitatively test for critical quality attributes (CQAs) of a vaccine product. While clinical (human) efficacy of a vaccine can never be predicted from pre-clinical evaluation of CQA, precise and accurate measurements of antigen content and a relevant biological activity (termed “potency”) elicited by the antigen allow selection of potentially safe and immunogenic doses for entry into clinical trials. All available vaccine technology platforms, novel and traditional, are being utilized by different developers to produce vaccines against SARS-CoV-2. It took less than a year from the publication of SARS-CoV-2 gene sequence to Emergency Use Authorization (EUA) of the first vaccine, setting a record for speed in the history of vaccine development. The largest ever global demand for vaccines has prompted some vaccine developers to enter multiple manufacturing partnerships in different countries in addition to implementing unprecedented scale-up plans. Quantitative, robust, and rapid analytical testing for CQA of a product is essential in ensuring smooth technology transfer between partners and allowing analytical bridging between vaccine batches used in different clinical phases leading up to regulatory approvals and commercialization. We discuss here opportunities to improve the speed and quality of the critical batch release and characterization assays.

## Introduction

The focus of this review is on vaccine lot or batch release assays that are essential to monitoring critical quality attributes (CQAs) and ensuring that high quality and well-characterized vaccine products are manufactured consistently. It is especially important in this fast-moving landscape of development that control on quality and consistency is maintained as manufacturing scale up and global supply chains are progressed. Analytical bridging through demonstration of CQA-based comparability between lots will be needed all the more in COVID-19 vaccine programs in order to minimize the need for time-consuming and expensive clinical bridging.

The portfolio of COVID-19 vaccines in development is large and expanding by the day, utilizing all available novel and traditional vaccine platform technologies. Among these, the mRNA platform has recently delivered the first two COVID-19 vaccines, which also happen to be the first ever vaccine products out of this technology platform^1–4^. In addition, the viral vector platform, which earlier yielded two vaccines against Ebola, using both replicating and non-replicating vectors^5–7^, has now delivered vaccines against SARS-CoV-2^8–12^. Additional platforms being used include live attenuated viruses (LAV), inactivated viruses, and recombinant proteins and protein-based virus-like particles (VLPs), all of which have a long history of delivering approved vaccines against other viruses. Nevertheless, there are opportunities for implementing faster, more sensitive and robust batch release assays.

Interim analyses of data from phase 3 trials of mRNA vaccines have indicated excellent efficacy^13,14^. Within the mRNA platform, self-replicating or self-amplifying mRNA (sa-mRNA) constructs appear to offer the advantage of potential efficacy at a lower dose^15^. However, this has yet to be demonstrated especially in the context of SARS-CoV-2.

This review will outline opportunities to improve the speed of batch release testing without compromising quality. This is particularly important for vaccines against COVID-19, which will require a speedy release of vaccine batches to ensure urgent delivery. Robust and faster turn-around assays for potency and other selected CQA will also be important for monitoring long-term and accelerated stability. The requirements of toolboxes and assays are vaccine platform and product dependent, although there are commonalities. In particular, we will focus on potency assays, which are key to delivering safe and immunogenic doses of vaccines. Although assays are established for the proven platforms, such as LAV and recombinant proteins, faster and more robust in vitro assays can be developed for some CQA.

In addition to antigen, the final formulation of the vaccine drug product (DP) often contains adjuvants and excipients such as stabilizers or cryo-protectants. Batch release testing for DP must include key tests for these components. Furthermore, any potential interference of these components in antigen assays, e.g., potency, must be ruled out or addressed.

As of January 25th, 2021, according to CEPI’s ongoing landscape analysis^16,17^, approximately 58 candidate vaccines globally are in different phases of clinical trials and, additionally, several are very close to entering phase 1 human trial. Sixteen candidate vaccines are already in phase 2b/3 trials, while rest of them are in 1, 1/2, and 2 clinical phases. In the coming weeks, some of these data points may possibly change because of recent emergency use approval (after phase 3 completion) of a few vaccines, which may enter post licensure phase 4.

Over the years, regulatory agencies such as the Food and Drug Administration (FDA) of USA and the European Medicines Agency (EMA) as well as the World Health Organization (WHO) have provided guidelines and recommendations for quality control of vaccines produced by different platform technologies. General guidelines for vaccines against COVID-19 have been published^18–25^, and more specific recommendations are currently being drafted.

### Principles of analytical evaluation based on CQA

CQA-based assays are, for the most part, product and platform specific. Additional critical assays, e.g., residual host cell and process-related impurities such as DNA, proteins, DNAase, trypsin, and serum albumin, may be generic but have impact on product safety and integrity. Quantitative and sensitive methods are well developed for such impurities and will not be discussed here. Microbiological testing including sterility is crucial to ensuring safety of any product. Traditional, culture based, sterility testing requiring a couple of weeks is often the slowest and rate-limiting step in vaccine lot release. Rapid and reliable sterility testing methods have been reported but, as of now, not received more than a limited degree of regulatory acceptance for the release of short shelf-life cell therapy products^26^. These may be further evaluated in the context of the faster batch release of vaccines against COVID-19.

Potency assays for LAV vaccines have traditionally utilized cell culture based infectivity testing, such as Median Tissue Culture Infectious Dose (TCID50) and plaque assays, which, depending on the virus, often take several days to produce definitive indication of cytopathic effects (CPE). These CPE-based methods are also being used for viral vectored vaccines. Alternative methods with increased sensitivity of detection, while keeping the essential cell incubation at the front end, have been extensively studied. These methods can significantly reduce turn-around time and offer higher throughput. In terms of rapid development and delivery of vaccines against SARS-CoV-2, there are now urgent needs and opportunities for implementing such assays with rigor and with a view to regulatory acceptance. It is possible and, indeed, highly desirable to combine speed with quality as well as to implement innovative analytical methods that will also improve precision and accuracy.

**Fig. 1:**
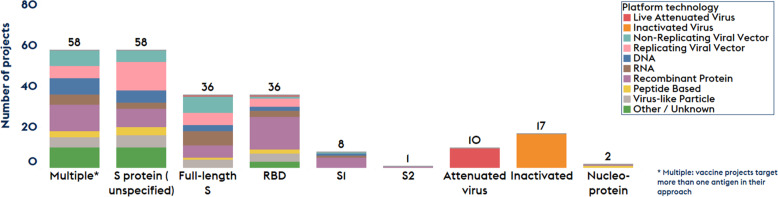
Landscape of target antigens included in COVID-19 vaccine development. The number of projects is shown for each target antigen. Different platform technologies being used are coded with different colors as defined in the inset.

It appears that the majority of the developers have selected either the full-length spike glycoprotein (S-protein) or a part of it, such as the angiotensin converting enzyme-2 (ACE-2) receptor binding domain (RBD) as antigen, while over 50 vaccine candidates also included more than one target, e.g., S, M, E, and N proteins (see Fig. 1). For all nucleic acid- and viral vector-based projects, expression of the protein antigens corresponding to the respective transgenes should ideally be tested in appropriate cells as a part of potency assays. Antibodies raised in the sera of vaccine recipients are expected to contain SARS-CoV-2 binding and neutralizing antibodies. Published data suggest that the S protein could induce neutralizing antibodies^27^.

The quality of a vaccine must be evaluated by analytical methods that reflect its identity, purity, structural integrity, and biological activity as a measure of potency. These measurements and quantitative determination of doses to be delivered must be as accurate and precise as possible, though the particular assays for quantifying these CQA depend on the platform and the product being administered to the vaccine recipients. The in vitro potency assay, as one example, will vary for different technologies (see Table 1). Even for one developer using a particular platform, multiple sites around the globe will be used to enable the manufacturing and rapid distribution of hundreds of millions of doses in different parts of the world. Thus, setting and meeting specifications for quality indicating assays are of paramount importance in ensuring delivery of safe and effective vaccines of consistent quality worldwide.

**Table 1:** Analytical methods typically used for in vitro vaccine potency determination.

Platform	Recombinant protein	Inactivated virus	LAV	Viral vector	mRNA	pDNA
Content	Optical density	Single-radial-immunodiffusion (SRID)	RT-PCR (qPCR, ddPCR)	DNA hybridization	Optical density	Optical density
	BCA	ELISA	Nanoparticle Tracking Analysis (NTA)	RT-PCR (qPCR, ddPCR)	RT-PCR	RT-PCR
			HPLC	Optical density	Fluorescent nucleic acid stain	Fluorescent nucleic acid stain
				Nanoparticle Tracking Analysis (NTA)	ELISA	
				HPLC		
Transgene expression	Immunostaining	n/a	n/a	qPCR/Spectroscopy	Transcription assay	Transcription assay
	Affinity			ELISA	ELISA	ELISA
				Flow cytometry	Flow cytometry	Flow cytometry
Functional viral titer (Infectivity)	n/a	n/a (However, loss of infectivity should be shown)	TCID50	TCID50	n/a	n/a
			Plaque assays	Plaque assays		
			ELISA	RT-PCR (qPCR-based potency assay - QPA)		
			Flow cytometry	ELISA		
			Fluorescent focus assay (FFA)	Flow cytometry		
				Fluorescent focus assay (FFA)		

There is significant expertise among many developers in analytical assays for batch release and characterization in the context of developing vaccines against pre-COVID-19 pathogens, using the same technology platforms. However, it is of utmost importance to focus on implementing those assays and tools that will be the most direct and precise indicators or surrogate measures of safety, potency, and immunogenicity. The development and execution of these methods may be dependent on the clinical phase. The critical assays such as those mentioned above should be qualified by the time of clinical entry (phase 1/2a) and, ideally, validated before phase 3. Development of reproducible, scientifically sound assays and standards during the pre-clinical phase allows efficient process optimization, facilitates regulatory interactions and entry into clinic.

In vitro methods are typically preferred for Chemistry, Manufacturing, and Control (CMC) batch release as they are more precise and robust than in vivo assays and have much shorter turn-around time. However, correlation between in vitro relative potency (IVRP) and in vivo immunogenicity (in a relevant animal model) may be desirable as a background rationale for a potency assay. Development of this correlation in a dose-dependent manner may be complementary to developing immunological assays that can detect and quantify virus-binding as well as virus-neutralizing antibodies in animals and, later, in clinical (human) sera samples.

### Analytical comparability

CQA-based evaluation of lot-to-lot comparability is an important component of CMC activities. This ensures that vaccine lots being used for successive phases of clinical trials are equivalent based on key CQA of the product such as potency, purity, and physical chemical integrity. Maintaining comparability between smaller scale material such as that often used in pre-clinical toxicity study and phase 1 trial with later phase clinical trial material (CTM) produced by larger scale processes is a regulatory requirement. Any process modification and formulation change between these phases, such as addition of an approved stabilizer, would also need to be substantiated by CQA-based comparability. This is a part of Good Manufacturing Practice (GMP) and provides a safeguard against potentially costly and time-consuming clinical bridging. Demonstration of comparability between phase 3 and commercial lots is required and is especially critical if scale up or scale out is involved, even though drug substance (DS) and drug product (DP) processes were locked prior to phase 3.

Comparability analysis may have an additional dimension for COVID-19 vaccines because, even for a single product, technology transfer between a developer and a manufacturing partner with higher capacity will have to happen in many cases in order to meet large global demands. The establishment of a clear plan between partners involved, aided by appropriate regulatory advice, will allow ensuring comparability between processes and product batches.

### Examples of CQA-based assays

A “potency assay” for a vaccine is, in reality, a biological activity assay that is a surrogate for immune response to be elicited by the antigen. This is typically a product and/or platform-dependent assay. While potency is intricately related to dose, quantitative methods to measure these attributes may be depending on the platform. For example, for a recombinant protein antigen, dose (or content) measurement is typically made by a quantitative protein assay or near-ultraviolet (UV) absorbance, while potency is measured by an immunoassay, at a series of pre-determined doses, as a surrogate of biological immune response. For LAV vaccines, on the other hand, both dose and potency can be expressed as infectious titer, although the content that a vaccinee is dosed with also contains non-infectious or defective viral genomes. In this case, the total number of viral particles or genome copies should be measured to track the ratio of infectious to total viral titer. For inactivated virus vaccines, the extent of inactivation is measured by following loss of infectious titer, while an immunoassay against a key epitope of the virus, which is still able to bind a specific mAb or polyclonal sera, can be used as a dose as well as a potency assay. For antigens inserted genetically in viral vectors, infectious titer of the vector has often been used as dose as well as potency, although the total number of viral particles has been reported as dose in other cases. In addition, expression of the target antigen encoded by the inserted gene in an appropriate cell line is expected as a potency assay. For DNA and RNA antigens, dose is readily measured by absorbance and fluorescence methods, or by quantitative PCR (qPCR). However, potency should be separately measured by transfection of appropriate cell lines and expression of the protein antigen.

Potency is also a primary stability indicator and will need to be monitored as a function of time for all candidate antigens in bulk (DS) as well as formulated DP. Other stability indicating CQA includes physical, chemical, and structural integrity. Aggregation, degradation or structural unfolding can cause loss of biological potency and may trigger toxicity issues, e.g., by inducing undesirable immune response. Depending on the nature of the antigen, i.e. the technology platform, these assays will be different, but the same basic principles apply.

### Recombinant protein-based vaccines

For subunit and VLP antigens and, indeed, for all classes of antigens, a dose-dependent correlation between in vitro potency and immune response in animal models often forms the basis for a potentially efficacious and safe dose selection in clinical trials. However, as a lot release assay, an in vitro potency assay is typically preferred for a few reasons including higher precision, lower inter-assay variability, faster turn-around and higher throughput. Indeed, this appears to be the preference among developers and regulatory agencies for vaccines being developed against SARS-CoV-2. For antigens that work predominantly by a humoral pathway, immune response or “immunogenicity” can be determined by measuring levels of antibodies in animal sera that bind to target epitopes on the antigen. Immunoassays such as binding and competitive enzyme-linked immunosorbent assays (ELISA), and Surface Plasmon Resonance (SPR) are typically employed. T-cell mediated responses or Cell Mediated Immunity (CMI) is determined by evaluating induction of cytokines such as interferons (IFN), interleukins (IL), and tumor necrosis factors (TNF). Further pre-clinical work, outside of CMC, may include testing for neutralization of the whole virus at a relevant titer by antibodies raised in animal sera.

There are several examples of recombinant protein-based subunit and VLP antigens for which in vitro potency assays have been successfully developed. For example, for approved VLP vaccines against hepatitis B virus (HBV) and human papilloma virus (HPV), the correlation between in vitro ELISA and in vivo production of neutralizing antibodies have been well-established^28–30^. IVRP assays have been accepted by regulatory agencies. For a trimeric post-fusion F-protein-based Respiratory Syncytial Virus (RSV) vaccine, which was in clinical development (but not an approved product), the correlation between a sandwich ELISA IVRP and in vivo immunogenicity was established^31^, and IVRP was used as a lot release potency assay.

While ELISA, in various forms such as direct binding, competitive, or sandwich, has been a widely used and reliable technology, newer immunoassay technologies provide fast turn-around, high throughput, and good precision. An example is VaxArray, introduced by InDevR^32^. In addition, SPR and Bio-Layer Interferometry (BLI) are alternative options for direct antigen-antibody binding assays^33,34^. The mAb CR3022, originally developed against the S-protein of SARS-CoV, also binds with high affinity to SARS-CoV-2 (although not neutralizing) and can be used for ELISA or any of these alternative immunoassays as a lot release potency assay. SARS-CoV-2 S-protein RBD specific mAbs have been reported^27^, and several are commercially available. A surrogate SARS-CoV-2 virus neutralization assay has been published, which is based on antibody-mediated inhibition of interaction between S-protein and angiotensin converting enzyme-2 (ACE-2) receptor^35^.

The reliability of a potency immunoassay depends on the accuracy and precision of an independent assay to measure the content (dose) of the antigen being used. For recombinant protein antigens, spectrophotometric methods (such as absorbance at 280 nm or A_280nm_) or a dye-based protein assay may be used. Measurements of A_280nm_ should include corrections for light scattering that may result from protein aggregation.

Physicochemical and structural integrity of protein antigens are other CQA that can affect both potency and safety. Preservation of key epitopes can be determined by binding of specific mAbs, which can also give an indication of overall integrity and stability of the tertiary structure. Direct measurements of stability of secondary structures and thermal unfolding of protein antigens can be monitored by far-UV circular dichroism (CD) and differential scanning calorimetry (DSC). These are relatively quick and supportive tools, although not of high resolution. As a part of extensive higher order structural characterization of, cryo-electron microscopy was performed on a candidate antigen, NVAX-CoV2373, which is a stabilized form of pre-fusion spike glycoprotein^36^. During development, leading up to a final process, detailed structural characterization provides a strong foundation for structure-function correlation for protein antigens. Once this foundation is established, a simple functional assay (e.g., antibody binding) that consistently correlates with structural perturbation can serve as a surrogate CQA assay. In addition to initial quantitation of the level of purity, protein degradation and post-translational modification at the primary structure level should be watched for and integrated in the stability program. While VLPs constitute self-associated protein units of well-defined sizes, the possibility of non-specific protein aggregation should be monitored by well-described hydrodynamic methods, e.g., light scattering based techniques. Aggregation of HPV 11 and HPV16 L1 VLPs was directly correlated to loss of potency as measured by antibody production in mice^37^. Non-specific aggregation can potentially also cause undesirable immune response.

Historically, recombinant protein-based vaccines have always required adjuvants for optimal immune response. Aluminum salts such as aluminum hydroxide, phosphate, and hydroxy-phosphate-sulfate have been the only approved adjuvants for decades. In recent years several new adjuvants, based on lipid A such as monophosphoryl lipid A and glucopyranosyl lipid A in a stable emulsion (MPLA, GLA-SE), squalene (AS03, MF59), and saponins (Matrix-M), have been approved in vaccine products or for clinical trials. Another example is Cytosine phosphoguanine (CpG) synthetic DNA. The current pipeline of candidate vaccines against SARS-CoV-2 includes stabilized pre-fusion form of the surface protein in combination with adjuvants such as AS03, CpG 1018, MF-59, and Matrix-M^38–41^. In these cases, vaccine DP release will need to include purity profiles of such adjuvants.

### Nucleic acid-based vaccines: pDNA and mRNA platforms

#### Commonalities and differences

Nucleic acid-based vaccines code for selected protein antigens and rely on human cells to produce these antigens after administration. Both pDNA and mRNA technologies are being used in the development of vaccines against SARS-CoV-2, based on nucleotide sequences that would express the S-protein in human cells. Neutralization by antibodies raised in animal sera as well as protection against virus challenge in vaccinated animals were used to support selection of potentially safe and immunogenic dose for initial clinical trials. In the early phases of clinical trials (phase 1/2a), it may be sufficient, from a regulatory perspective, to move forward with a CMC package containing CQA-based tests such as genetic identity, conformational purity, and content. This could be supported by the demonstration of antigen-specific immune response in animal models using a serological assay such as the plaque reduction neutralization test. However, in phase 3, a potency assay is expected to demonstrate the expression of the protein antigen in a relevant cell line and be correlated with in vivo expression. WHO guidelines and regulatory documents from EMA and FDA have recently provided these recommendations^42–44^. Also, a guideline listing appearance, identity, potency, and integrity analyses for Official Control Authority Batch Release (OCABR) of mRNA vaccines has been published^23^.

Antigen expression in transfected cells can be demonstrated qualitatively, e.g., by Western blot analysis using antibodies against SARS-CoV-2. During vaccine development efforts targeted at SARS-CoV, this was accomplished for an experimental DNA vaccine that utilized the nucleoprotein (N-protein) coding sequence^45^. Quantitative determination of the levels of antigen can be obtained by use of fluorescently labeled SARS-CoV-2 S-protein antibodies for pDNA (and mRNA) projects that are using the coding sequence of this antigen. Flow cytometry has been used for quantitative detection of antigen expression in nucleic acid-based vaccine candidates^46^. Transgene expression of pDNA vaccines may also be quantified at the RNA level in transfected cells by RT-PCR^47^.

Transfection efficiencies, as a measure of potency of mRNA constructs, in dendritic cells and several other cell lines have been determined using fluorescently labeled mRNA and flow cytometric detection^48^. Examples include mRNA transcripts encoding Rabies and Zika antigens^49,50^.

There is a fundamental difference in the mechanism of action between pDNA and mRNA antigens. This leads to several important differences in the characteristics of the resulting products. pDNA antigens require intracellular transport to accomplish transcription in the cell nucleus, followed by exit into the cytosol where translation happens. mRNA antigens, on the other hand, are delivered directly into the cell cytoplasm for translation to the corresponding protein. This difference is the most likely reason why required doses for pDNA vaccines are typically much higher than those of mRNA vaccines. pDNA vaccines are best delivered by electroporation using a special device after intramuscular or subcutaneous injections. The device requires regulatory approval, but once approved, can be used for electroporation of all pDNA antigens unless changes are made in its design. pDNA antigens usually have higher stability and formulation requirements are simpler, while mRNA antigens are typically less stable physically and require encapsulation by lipid nanoparticles (LNP) to protect them from degradation by RNAses. This also results in a relatively larger number of release and characterization testing for mRNA-based DPs.

#### Bioanalytical advances in mRNA platform

Self-replicating RNA or sa-mRNA is a further development of the mRNA platform with potential to achieve equivalent immune response at a lower dose than needed for standard mRNA^51^. An in vitro potency assay for a sa-mRNA construct was developed to measure replication efficiency by capturing intermediate double-stranded RNA (dsRNA) and comparable protein expression in individual cells by an antigen-specific antibody. This assay is based on the indirect antibody labeling approach where protein expression proficiency measured by transfection of antigen encoded mRNA into Baby Hamster Kidney (BHK) cells followed by staining with fluorochrome tagged primary antibody^52^. The frequencies of fluorescent positive cells indicate the level of protein expression, confirming antigen identity within 48 h. This procedure could easily allow detecting multiple proteins in a single cell and could be adaptable as a platform-based approach by using secondary antibodies to detect multiple antigen-specific antibodies. Intracellular dsRNA produced during the replication cycle is a marker of RNA amplification. BHK cells transfected with influenza and Zika virus sa-mRNA reported high frequencies of dsRNA positive cells stained (as measured by flow cytometry) with anti-dsRNA antibody, unveiling launch of self-amplification^52,53^. Interestingly, immunogenic profile of Zika virus sa-mRNA vaccine was found to be comparable with frequencies of dsRNA positive BHK cells and protein expression^53^. Similarly, direct and sandwich ELISA could also be developed using electroporated cells with RNA vaccines. The current portfolio of mRNA-based COVID-19 vaccine candidates includes sa-mRNA candidates.

Developers of mRNA vaccines have paid close attention to stability and efficient delivery of antigens. As capping at the 5′-end may have an impact on stability and translation, the percentage of capped RNA is a CQA that should be measured quantitatively. Methods typically employed for this purpose have been reviewed^48^. For drug product formulation and delivery, LNPs have been extensively used with the objective of stimulating innate immune response, and these studies include clinical trials^54^. More recently, mRNA transcripts encoding for pre- and post-fusion RSV F-protein and formulated in LNP have been found to elicit protective immune response in rodent models^55^. These formulations typically involve encapsulation of mRNA in LNP. Therefore, encapsulation efficiency should also be determined as a CQA. The mean hydrodynamic size and size distribution (polydispersity) of LNP should be kept within target specification range. Dynamic or multiangle light scattering techniques are suitable for monitoring these parameters. The amount and purity of each lipid used in the LNP need to be monitored as these parameters are likely contributors to effectiveness and safety. Formulation of two candidate sa-mRNA vaccines against Zika virus with a cationic nanoemulsion (CNE) has been reported to induce potent immunity in mice and non-human primates^53^. In this case, a simple mixture of the mRNA antigen with the CNE adjuvant was used.

Removal of dsRNA in the final product resulting from in vitro transcribed mRNA transcripts, as completely as possible, is important as dsRNA is known to cause undesirable local, injection site, immune response. A facile method for the removal of dsRNA has been reported^56^. This is another CQA that should be quantitatively monitored as it relates to safety.

In the context of the physical stability of mRNA-based vaccines, significantly improved thermal stability was achieved for a freeze-dried, candidate Rabies glycoprotein mRNA vaccine^57^. Genetic stability should also be monitored over time during storage.

#### Recent deliveries of COVID-19 vaccines from mRNA platform

mRNA-based technologies constitute a relatively new platform. Two of the modified mRNA-based vaccines against SARS-CoV-2 have advanced rapidly through clinical phases^58–61^, and have recently delivered products that have received Emergency Use Authorization (EUA) by several regulatory agencies. These products, BNT-162b2 and mRNA-1273, were developed by Pfizer-BioNTech and Moderna Therapeutics, respectively. Both are based on non-replicating mRNA sequences encoding the full-length S-protein, packaged in LNP to provide protection against RNAses. The EMA Assessment Report on BNT162b2 provides a comprehensive summary of tests that were performed on this product^3^. Product-specific tests include in vitro bioanalytical batch release and characterization assays for the antigen and LNP.

### Viral vector-based vaccines

Viral vectored vaccines constitute a significant part of the global portfolio with 60 vaccine candidates being developed against SARS-CoV-2. Candidate vaccines are based on replicating and non-replicating vectors including, but not limited to, vesicular stomatitis virus (VSV), measles, modified vaccinia ankara (MVA), adenovirus 26 and 5 (Ad26, Ad5) and chimpanzee adenovirus Oxford construct 1 (ChAdOx1). These vaccines are being evaluated either in a homologous or a heterologous prime-boost regimen, or in a single dose regimen. Prior to COVID-19, two products, both against Ebola, using viral vector platforms have been licensed. In addition, clinical trials demonstrating acceptable safety profiles have been reported for all of the above-mentioned vectors^62–66^. Assessing safety of a viral vectored vaccine is based on the same principles as those applied to LAV. In addition, it is necessary, from a CMC standpoint, to monitor the concentration of replication competent viral particles that may appear during the manufacturing of a replication-deficient viral vector. The potency of a viral vectored vaccine should ideally reflect both infectivity and transgene expression^67–70^. The European pharmacopoeia expert group (Group 15) has provided guidance on appropriate analytical strategies for viral vectored vaccines^71^ and, in addition, OCABR guidelines have been published for analyses of non-replicating human and chimpanzee adenovirus vectored vaccines^21,22^.

Viral vectored vaccines are typically dosed based on infectivity and/or total viral particles. Assays to determine infectivity of viral vectored vaccines are based on the same cell-based principles and experience the same challenges as described below for LAV. Of the currently approved products for ebola, the recombinant VSV-based vaccine against the Zaire strain of ebola virus (rVSV-ZEBOV) utilizes a combination of infectivity determined by TCID50 assay and total viral particles assessed by droplet digital polymerase chain reaction (ddPCR) assay^72^. Similarly, the Ad26.ZEBOV/MVA-BN®-Filo heterologous prime-boost Ebola vaccine prime is dosed on the basis of total viral particles determined by qPCR and qPCR-based potency assay (QPA). QPA combines qPCR with a tissue culture-based infectivity assay to quantify the adenovirus (prime) potency, while the boost MVA-BN®-Filo potency is determined solely on infectivity through a flow cytometry-based method^73^. The throughput and turn-around time of the latter infectivity assays are improved compared to more classical TCID50, because the higher sensitivity of detection allows reduction of cell-virus incubation time from 7 days or longer to 2 days. Furthermore, accuracy and precision of analysis are also improved, and qPCR allows automated analysis^74^.

Quantifying virus particles may also be done using various particle sizing instruments. For instance, Nanoparticle Tracking Analysis (NTA), e.g., NanoSight^®^ (Malvern Instruments Ltd) and ZetaView^®^ (Particle Metrix GmbH), tracks the Brownian motion of particles through a flow cell in real-time. From the video parameters such as particle size and number are determined that can be correlated to VP quantity^75^. Another rapid analysis method for viral particle quantification is high-performance liquid chromatography (HPLC) where the column allows separation of intact virus particles from other cellular contaminants or fragmented virus particles^76^.

For adenovector-based vaccines, which are being widely used for COVID-19 response, absorbance measurements are commonly used for quantitation of virus particles, using methods based on the correlation between the protein content of adenovirus preparations and absorbance at 260 nm (for viral DNA) with appropriate controls^77,78^. The use of such methods enables in-process controls of adenovirus particle concentration and high throughput analysis for release. In addition, there are several commercial kits available utilizing the production of viral hexon proteins to analyze infectious titers of adenoviral stocks in 48 h.

Initially, semi-quantitative assays such as gel electrophoresis and Western blot are accepted to show the transgene expression of viral vectored vaccines, although for late-stage clinical trials and licensure more quantitative methods will be required to assess potency. A variety of methods may be applied, including biochemical (e.g., protein binding, enzymatic reactions), immunochemical (e.g., flow cytometry, ELISA), and molecular (e.g., PCR, microarray). For instance, the Ebola vaccine licenced by Janssen vaccines uses ELISA to determine transgene expression in the adenoviral prime vaccine and flow cytometry for the MVA boost^79,80^.

As viral vectored vaccines are recombinant products the genetic stability is an important CQA to prove that the gene of interest, as well as the vector, is uncompromised. In general, this is tested in characterization studies prior to licensure and not for batch release. This is done by passaging the virus, normally several passages beyond what the manufacturing passage will be, and sequencing several subsequent passages using either Sanger or Next Generation sequencing (NGS).

#### Recent advances in viral vector-based COVID-19 vaccine delivery

Interim analysis of phase 3 clinical trial for AstraZeneca-Oxford’s ChAdOx1 based vaccine (AZD1222) have been published^81^, and this vaccine has received EUA in the UK and many other countries^82^. Phase 3 clinical trial was slowed down, especially in the U.S., due to an adverse event, although observed at an extremely low frequency and with no demonstrated linkage to the vaccine. This demonstrates the critical role of bioanalytical characterization to the fullest extent, although response in each individual cannot be predicted with 100% certainty even for a placebo injection. Interim results of Ad26.SARSCoV-2.S vaccine trials by Johnson and Johnson have been reported^83,84^. This vaccine has the advantage of refrigerator (2–8 °C) storage stability and offers the possibility of protective efficacy from a single dose. This vaccine has now received EUA by FDA^85^.

### Live attenuated virus vaccines

This platform has produced some of the most effective vaccines for decades and has a sound track record for safety as exemplified by global usage of the measles and rubella vaccine in infants. The older technology of attenuation by multiple passaging through cells and the more recent introduction of attenuation by genetic engineering are effective. However, safety concerns include reaching the balance between eliciting protective immune response and causing vaccine-induced infection. For engineered viruses, the absence of reversion mutants must be monitored and NGS is an effective tool in this regard. The issue of potency versus safety is best addressed by potency assays that are as accurate and precise as possible. Traditional infectious titer assays such as TCID50, which are widely accepted by regulatory agencies, often suffer from large standard deviations. Depending on the virus, these assays may have a long turn-around time due to the low sensitivity of CPE detection, which requires a large number of replication cycles for a sufficient percentage of the cell population to be killed by infection. As described above in the section on viral vector-based vaccines, PCR or fluorescence-based detection techniques provide more sensitive detection and, thereby, allows reduction of the incubation time with cells. Published examples of these applications to several LAVs include but are not limited to measles, rotavirus, VSV, and HIV-1^86–88^. Fluorescent focus assay (FFA), a fluorescent antibody-based infectious titer assay, offers the advantage of sensitivity, automated foci counting and relatively high throughput. Compared to plaque and TCID50 assays, infection of cells can be quantitatively detected earlier in the process instead of having to await cell membrane lysis. Therefore, infectious titer, as focus forming unit, can be determined significantly faster compared to a CPE-based assay. FFA is used as a batch release potency assay for an approved LAV influenza vaccine^89^. However, it is important to establish that focus formation is a result of infection and one is not observing simply a binding event that may not lead to CPE. Demonstration of concordance with plaque or TCID50 assay can be a part of the FFA validation process^90^. With appropriate controls in place, FFA could provide a sensitive and reasonably fast cell-based potency assay for SARS-CoV-2 LAV projects. An emerging label-free technology for infectious measurements is based on laser force cytology to detect and quantify virus-infected cells. It appears to be more sensitive and much faster than the TCID50 assay, and may offer a higher precision^91^. This method can also be used for rapid in-process testing during manufacturing.

### Non-clinical and clinical assays

Outside of CMC, specific assays are developed in the pre-clinical and clinical trial phases to test immune response induced in animals and humans, respectively. These assays are further tested for robustness, qualified and validated in later stages to support the vaccine development process. Often, challenges including variability in in vivo biology and technical differences between laboratories can make comparison of data produced by individual laboratories in late phases and multicenter trials difficult. Additionally, multiple assays developed for vaccines of various modalities also augment complexities. To mitigate some of these challenges to reliable immunological profiling of each vaccine candidate based on different platforms, and to provide robust assays to facilitate the regulatory process, CEPI has established a global network of seven Central Laboratories (CLs). Immune responses elicited by different vaccines during the pre-clinical and clinical phases will be analyzed by implementing common protocols, antibody standards and equivalent key reagents across these laboratories^92,93^.

The antibody-mediated immune response relies on B-cell recognition of antigens and the release of antibodies. ELISA is often used to evaluate humoral responses. CEPI selected ELISA methods that exploit and capture signals of full-length SARS-CoV-2 S, RBD, and N-protein IgG antibody/antigen interactions. ELISA only measures binding antibodies while detection of functional antibodies is critical for vaccine efficacy and regarded as the golden standard. Robust pseudovirus and wild-type virus-based neutralization assays have been developed to detect neutralizing antibodies to SARS-CoV-2, as they are designed to detect antibodies capable of inhibiting viral replication. These assays are either validated or qualified.

CMI response relies primarily on T-cells and is often assessed with enzyme-linked immunospot (ELISPOT) or flow cytometry. The CEPI supported ELISPOT is an immunostaining assay that focuses on a quantitative measurement of the frequency of the cytokine secreting T-cells. This ELISPOT assay allows the detection of specific TH1 (IFN-γ) and TH2 (IL-5) cytokines-producing T-cells in peripheral blood mononuclear cells (PBMCs) stimulated with SARS-CoV-2 peptides spanning full-length S protein. These cytokines have been detected frequently with individuals infected with SARS-CoV-2 and predict immune protection^94^.

Clinical assays are used to quantify immune responses generated in human subjects after immunization, while potency assays are designed to measure optimal doses and surrogate biological activities that would potentially result in desired levels of immune responses. In setting up a potency assay, it is desirable to have knowledge-based hypothesis regarding the mechanism of action of a vaccine that may correlate with a clinically relevant immune response. However, for SARS-CoV-2 vaccines correlation of protection in humans is not yet known, although protection in animal models has been demonstrated for some candidate vaccines^47,58^. While such indication of protective efficacy, typically obtained in a handful of animals, is certainly promising, it is not a guaranteed predictor of protective efficacy in humans. Still, it is very encouraging that early clinical trials have demonstrated that the doses selected on the basis of potency assays used for CMC batch release have yielded high levels of immune responses^58,95^. The development of non-clinical immunoassays could synergistically help in establishing functionally meaningful and qualitative potency assays.

Most of the SARS-CoV-2 vaccine developers are using ‘’in-house” assays, reagents and panel of reference regents. This makes it difficult to compare immune responses across different vaccines. Therefore, it is critical to standardize assays and reagents to support advanced phase clinical trials. To address this issue, CEPI has facilitated the development of a WHO endorsed international antibody reference standard^96^, and vaccine developers are encouraged to include this standard in immunological assays. Furthermore, CLs in CEPI’s global network of laboratoriess are using common key reagents including coating protein, SARS-CoV-2 virus strain, virus pseudoparticles, peptide pools for PBMC stimulation, panel of standards and controls. This dual approach enables impartial comparison of data generated across vaccine trials.

### Closing comments

The COVID-19 pandemic has galvanized vaccine development efforts globally with an unprecedented sense of urgency. Developers from biopharmaceutical industry, small and large, and academic laboratories have stepped up with a mission to end the current pandemic. All vaccine platform technologies developed to date, old and new, are being employed. Regulatory agencies have prioritized reviews of investigators’ applications seeking entry into and progression through clinical trials. At this time, the first two mRNA vaccines have been given to more than 25 million people under EUA. Four viral vectored vaccines have closely followed in getting approval for emergency use in different countries, as well as four inactivated coronavirus vaccines. As clinical trials for many more candidate vaccines are progressing to advanced phases involving tens of thousands of volunteers, quality attributes of vaccine candidates that may be critical to safety and immunogenicity are being evaluated by developers and stringently reviewed by regulators in a closely interactive manner. Based on the large body of data already gathered from human clinical trials and from ongoing vaccination with EUA approved products in several countries, it is clear that safety profiles of vaccines produced using newer platforms have been excellent. In order to meet the urgent demands of virtually the entire global population, analytical assays of high reliability and short turn-around times should be implemented for testing CQA of each product. Such assays have been described in the literature in the context of development or study of other vaccines and can be adapted for vaccines against SARS-CoV-2, for example, in measurements of biological activity or potency. Batch release assays should preferably be performed in vitro. This is not only appropriate for adhering to 3R principles, but also preferable for practical considerations including savings in time and reducing irreproducibility associated with animal-based tests, which often leads to costly and unnecessary rejection of good quality batches. Reagents for in vitro immunochemical and biochemical assays, including the mAb CR3022, RBD of the S-protein, and human ACE2 are now being made available to eligible developers in collaborative arrangements with PATH and NIBSC. Robust and faster assays for CQA will expedite technology transfers between manufacturing sites, which are required for many COVID-19 projects in order to meet large supply needs among all populations of the world. A well-designed CMC plan will also facilitate regulatory reviews and approvals. Furthermore, selected CQA-based bioanalytical evaluation methods, outlined in part in this review, will allow comparing consistency in quality between vaccine batches used in different clinical phases and forming a valuable bridge that will extend to commercial products.

## Data Availability

This review article does not contain any new data. All of the research and associated data that this review is based on are available in the public domain and these publications have been cited in this article under “References”.

## Code availability

No custome code or mathematical algoritm has been used in this article.
